# Laborem Box: A scalable and open source platform to design remote lab experiments in electronics

**DOI:** 10.1016/j.ohx.2022.e00301

**Published:** 2022-03-31

**Authors:** Camille Lavayssière, Benoît Larroque, Franck Luthon

**Affiliations:** Universite de Pau et des Pays de l’Adour, E2S UPPA, LIUPPA, Anglet, France; Universite de Pau et des Pays de l’Adour, E2S UPPA, SIAME, Anglet, France

**Keywords:** Hybrid education, Remote laboratory, Open hardware, 3D printing, Blended learning

## Abstract

Hybrid teaching (face-to-face and distance learning) enables students to better prepare and complete their courses. In science, technology, engineering and mathematics, it is important that practical training be an integral part of the curriculum. Laborem project developed at the technological university institute in Bayonne, France, enables undergraduate students to carry out part of their lab experiments in electronics remotely. Started in 2011, Laborem platform was based on proprietary solutions. Since 2017, the platform has migrated to open source software (PyScada) and open source interface box (Laborem Box), which was developed in order to enable the connection of several circuit boards to be studied. These boards, called plugs, are easily interchangeable and enable teachers to quickly adapt the proposed circuits to their course. The software also provides a simple front panel to adapt the human machine interface that is available for students. Laborem Box consists of a 3D printable box, a power supply board, a set of plugs, and a motherboard that enables students to study the selected plug. In addition, a single board computer is embedded and a hard disk can be used if necessary. This paper is intended to describe the hardware and software design of Laborem platform, and to serve as a guide to explain how to duplicate and deploy this system, primarily dedicated to undergraduate students for learning basic electronics.

## Graphical abstract

**Figure:**
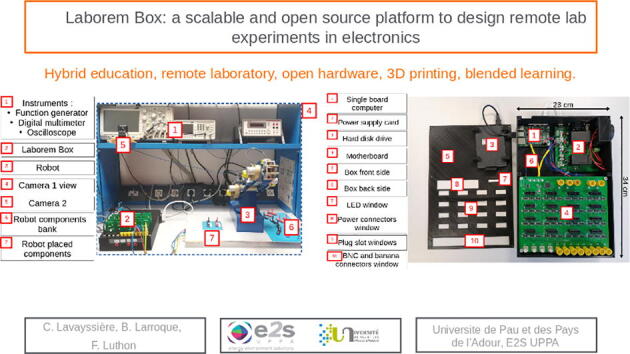

**Specifications table:**

**Table:** 

**Hardware name**	Laborem Box
**Subject area**	• Engineering and material science
	• Educational tools and open source alternatives to existing infrastructure
**Hardware type**	• Measuring physical properties and in-lab sensors
	• Field measurements and sensors
	• Electrical engineering and computer science
**Closest commercial analog**	https://labsland.com/en/labs/electronics-community
**Open source license**	GNU General Public License v3.0
**Cost of hardware**	429.03 €
**Source file repository**	https://doi.org/10.5281/zenodo.5564369

## 1 Hardware in context

One of the main objectives of distance laboratories is to extend the possibilities of practical experimentation in science, technology, engineering and mathematics (STEM) university degrees. Indeed, the profile of students in higher education is diversified and now well adapted to new information technologies. Moreover, as shown in [1], the massification of students in higher education has led to changes such as distance learning techniques through the use of massive open online courses (MOOCs), e-learning, and simulation. In order to address this issue, it is crucial to create new tools for teachers and learners, such as remote labs embedded in game-like scenarios [2].

Round-the-clock accessibility, improved learning processes, and practical examples for classroom instruction are among the many benefits that remote labs bring to teachers and students [3]. This makes distance laboratories well suited to the dynamic online experience of today’s students. A remote lab is different from a virtual lab, which is just a computer model that is less expensive and easier to implement and share, since it uses only simulation [4]. For over twenty years, there has been numerous publications that can attest to the massive use of remote labs as a learning technology [5]. For example, more than 30 websites are referenced on the Wikipedia web page “remote laboratory” [6].

To date, there has been no remote laboratory offering open source hardware like electronic boards developed to perform practical work. The Open Source Hardware Association (OSHWA) [7] defines Open Source Hardware (OSHW) as machines, devices, or other physical objects whose design has been made public so that anyone can make, modify, distribute, and use these objects. Among all remote laboratories and remote experiments listed in [3], only a small number use open source software (see Table 1). However it has been observed that there is a tendency to use low-cost existing material or even open source software to improve standardization and simplicity of use. Many remote education labs use single board computers (SBC), such as the Raspberry Pi, to control their equipment [8], [9], [10]. This enables for the design of low-cost platforms compared to a workstation or server [11]. In order to make electrical circuits and to connect several instruments, some still use breadboards [10] as we did before 2017, others design their own printed circuit boards (PCB) [11], [12]. Other laboratories use microcontrollers like Arduino, which can be programmed to acquire and generate electrical signals in order to perform a wide variety of tasks such as lighting, heating, robot control, embedded computing, etc [10], [11], [12], [13], [14], [15].

**Table 1:** Comparison of remote laboratories developments from [3].

Remote laboratory	Open source software	Open source hardware
UNILabs	Yes^a^	No
WebLab-Deusto LabsLand	Yes^b^	No
RexLab	Yes^c^	No
ISES RemLabNet	No	No
VirtualRemoteLab	No	No
GOLDi	No	No
OE@FEUP HTML/LabVIEW	No	No
e-lab IST	No	No
Stanford iLABS	No	No
iLAB LabShare	No	No
FarLabs	No	No
Laborem	Yes^d^	Yes^e^

a https://github.com/UNEDLabs

b https://github.com/weblabdeusto

c https://github.com/RExLab

d https://github.com/clavay/pyscada-laborem

e https://doi.org/10.5281/zenodo.5564369

The Laborem project developed at the technological university institute (IUT) in Bayonne, France, aims to offer remote electronics practical work via a software and hardware platform that is inexpensive, simple to deploy, open source, and scalable. The first version of Laborem was based on proprietary hardware and software, using National Instruments (NI) solutions including LabVIEW software [16] and Elvis Protoboard [17]. This solution was costly and complex to reproduce. However, it was efficient to test the proof of concept of a remote lab in our classes [2], [18].

**Fig. 1:**
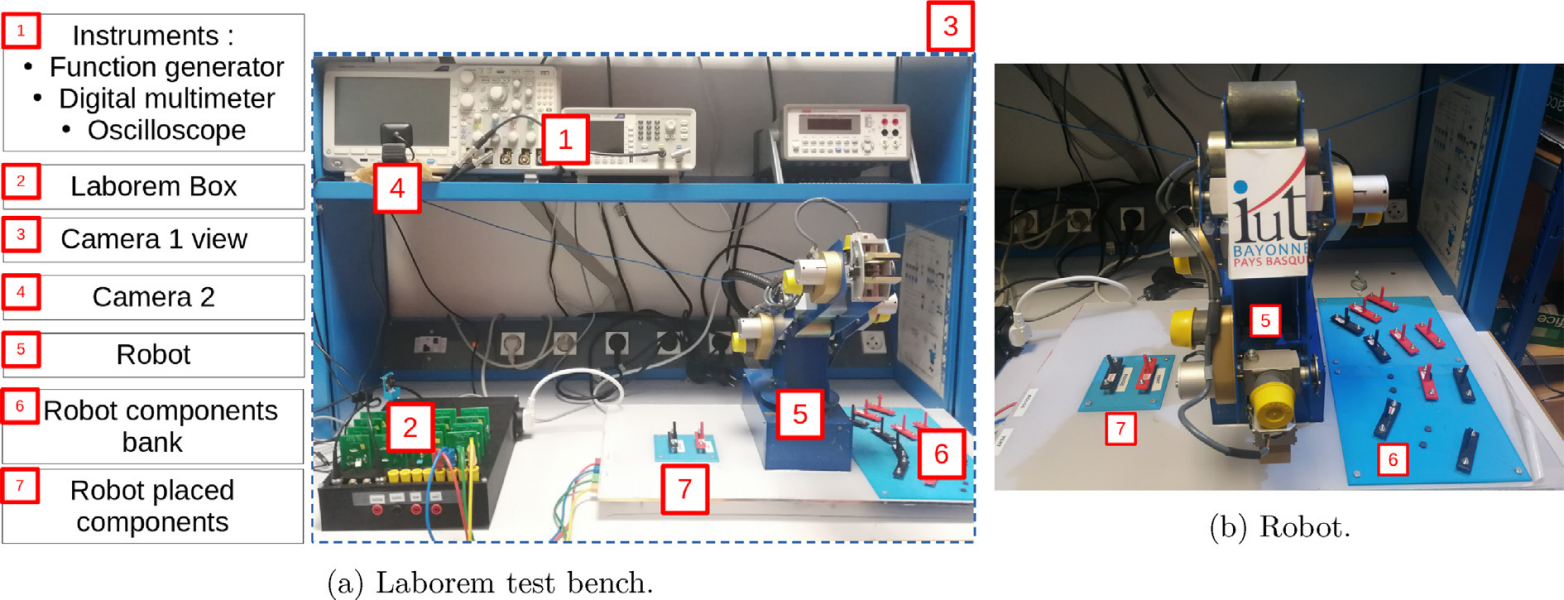
Overview of Laborem test bench.

The VISIR project is probably the most famous one enabling to follow distance learning courses in electronics. It is accessible through LabsLand^1^ or other platforms (see VISIR SIG^2^ for more information). Laborem does not enable the student to build his circuit from scratch like VISIR, but it does enable a teacher to easily propose new circuits. VISIR also enables the study of pre-assembled circuits such as “black boxes” [19]. One objective of Laborem is to maximize the motivation and immersion of the student by proposing a list of circuits, some of which are to be completed with the help of a robot (see Section 2.1 and Fig. 1) and and with the help of cameras enabling to visualize the platform (instruments, robot and Laborem Box, see Fig. 1). However, VISIR costs about 40,000 euros [3], which makes it different from the Laborem project whose hardware cost including the Laborem Box, a camera, the instruments and the robot is about 3500 euros.

**Fig. 2:**
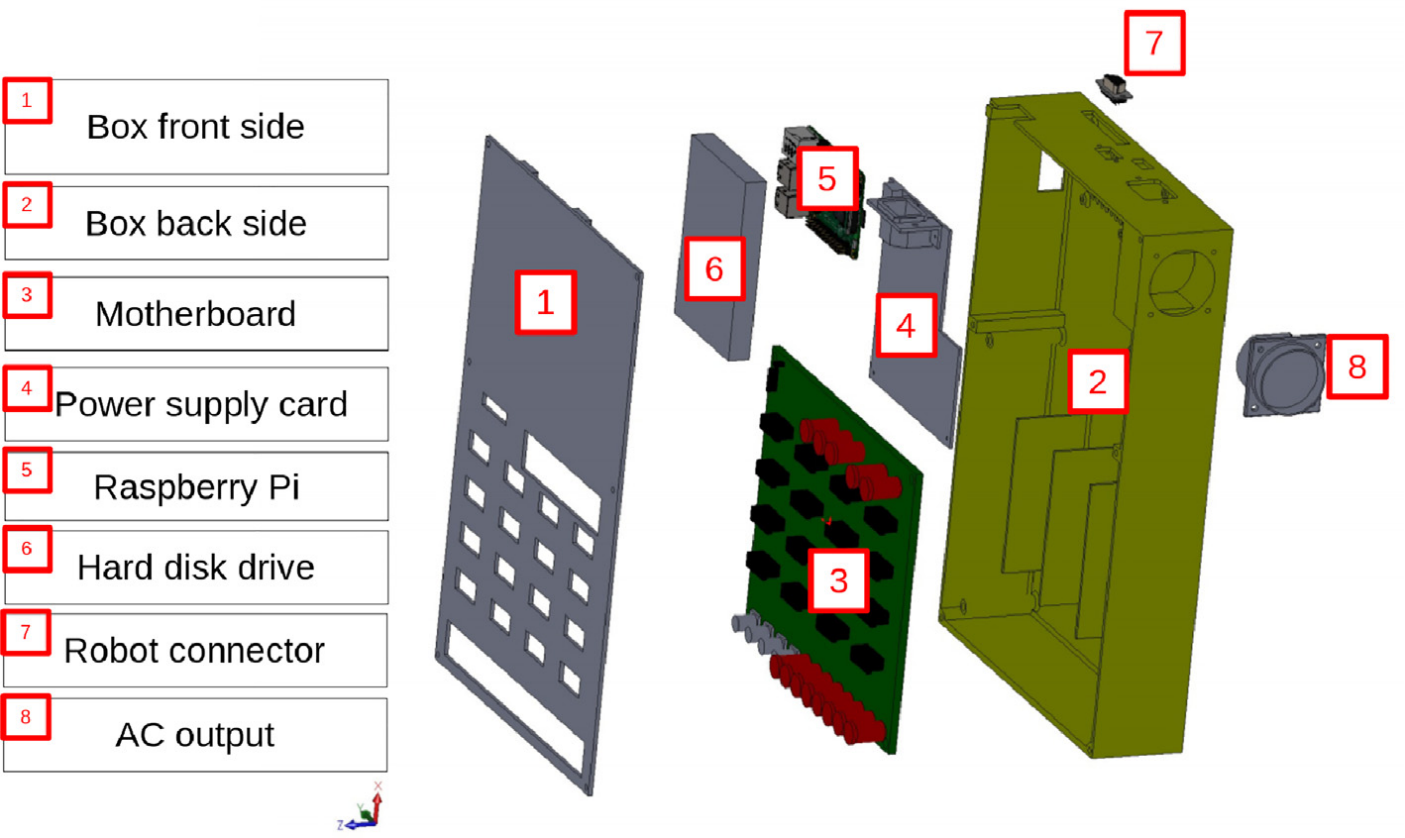
Overview of the Laborem Box.

Laborem Box, the hardware presented in this paper, consists of a 3D printable box, a power supply board, several removable electronic cards called plugs (devices under test) and a motherboard that can be connected to various instruments. These electronic circuits are easy to make according to the needs of each course. The place is also foreseen for a SBC and an optional hard disk (see Fig. 2).

The rest of the paper is organized as follows: Section 2 describes the hardware, Sections 3, 4 summarize the design files and materials required, Sections 5, 6 give instructions for building and operating the system, and finally Section 7 characterizes the use of the platform.

## 2 Hardware description

### 2.1 Overview

**Fig. 3:**
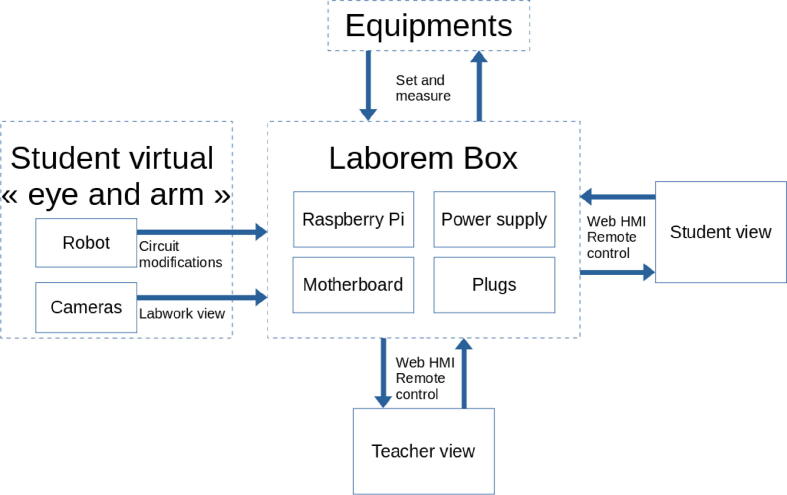
Overview of Laborem system architecture.

Since we decided to switch to open source software and, as much as possible, to utilize open source hardware, the Laborem’s architecture is centered on the Laborem Box (Fig. 3).

The Laborem Box is based on a server, which is here a Raspberry Pi, that hosts the human–machine interface (HMI) accessible through a web page by students and teachers. The HMI is reachable from any device (computer, tablet or smartphone) and any operating system using a modern web browser.

The power supply card is designed to provide all the voltage references mainly required for integrated circuits of the platform (switches, multiplexer, operational amplifiers).

The motherboard selects the appropriate device under test (DUT) by connecting all the I/O to the plug slot (see Section 2.3). All voltages are always connected to all plug slots.

Plugs are designed to offer a robust and easy-to-change system that enables the facility to build and study any electrical circuit.

An optional robotic arm (5 degrees of freedom (DOF)) is used for circuit modification [20], [21] (see the video^3^), and two cameras mimic the student’s eyes to see all the equipment of the workbench (see Fig. 1) which enables to follow the measurements in progress. The robot enables the teacher to propose incomplete circuits (with 2 missing components) that the student must choose using the available components from the bank of components (see Fig. 1).

The particularity of the board is to simply enable teachers to:

• Propose their electronic practical work to students via a remote access and a simple administration panel.
• Choose the instruments needed for the practical work.
• Connect specific instruments using BNC and Banana connectors.
• Create plugs necessary to study a set of electrical circuit.
• Change quickly the plugs to switch easily to other practical works.

The instruments currently connected to the Laborem Box use the Standard Commands for Programmable Instrumentation (SCPI) and the Virtual Instrument Software Architecture (VISA) (now managed by the IVI foundation^4^). Instruments are connected to the Raspberry Pi via GPIB or USB. Any instrument using a protocol managed by PyScada can be used (for example: VISA, Modbus, I2C, OneWire, BACnet, OPC-UA, WebService, Serial). This list is updated over time.

### 2.2 The 3D box

**Fig. 4:**
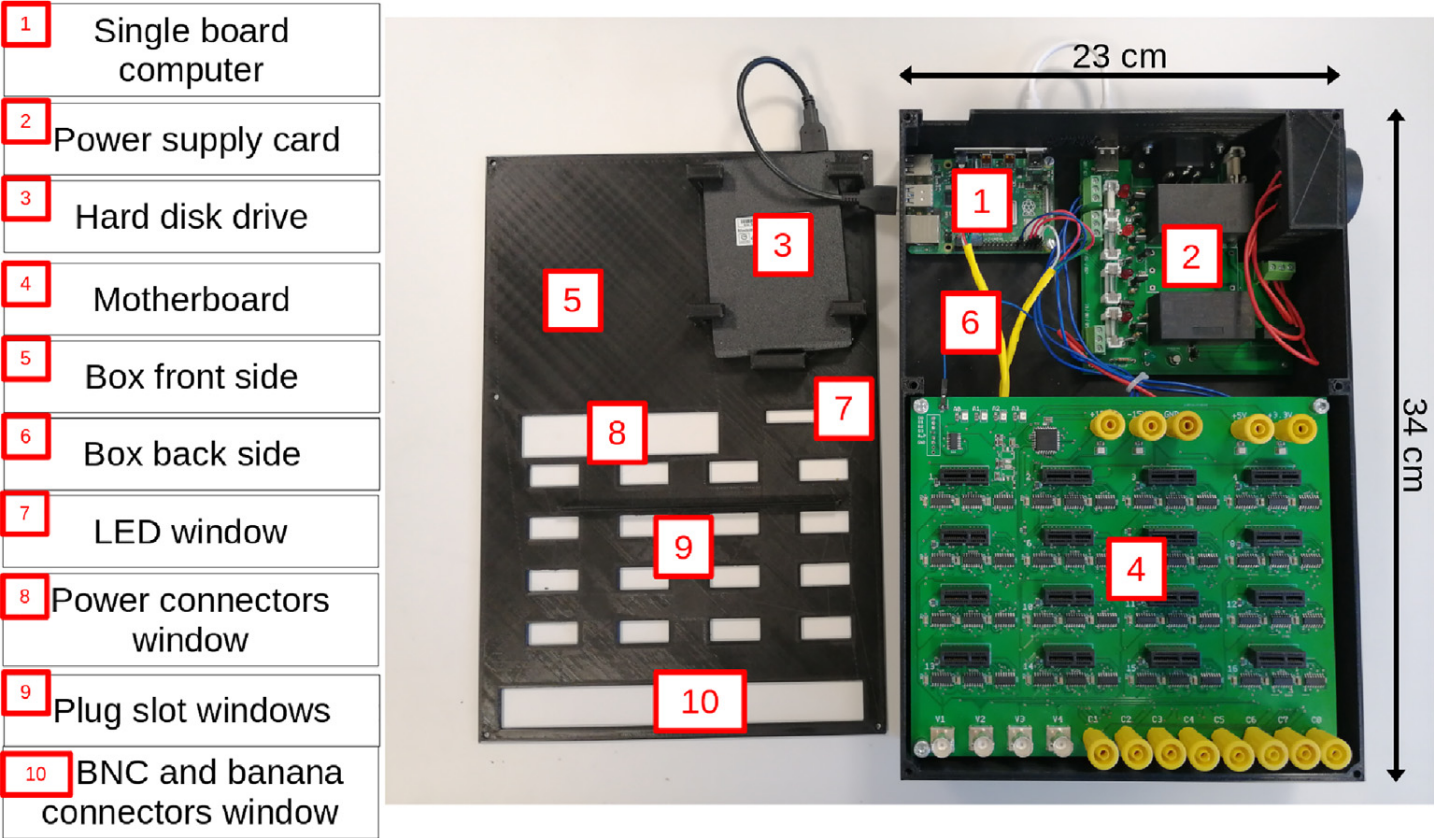
The 3D box.

The 3D box, consisting of two parts (front and back side), is designed to hold the motherboard, the power supply, the SBC, and an optional hard disk (see Fig. 4). It secures the installation and should only be opened when the system is powered off and all cables are disconnected.

### 2.3 The motherboard

**Fig. 5:**
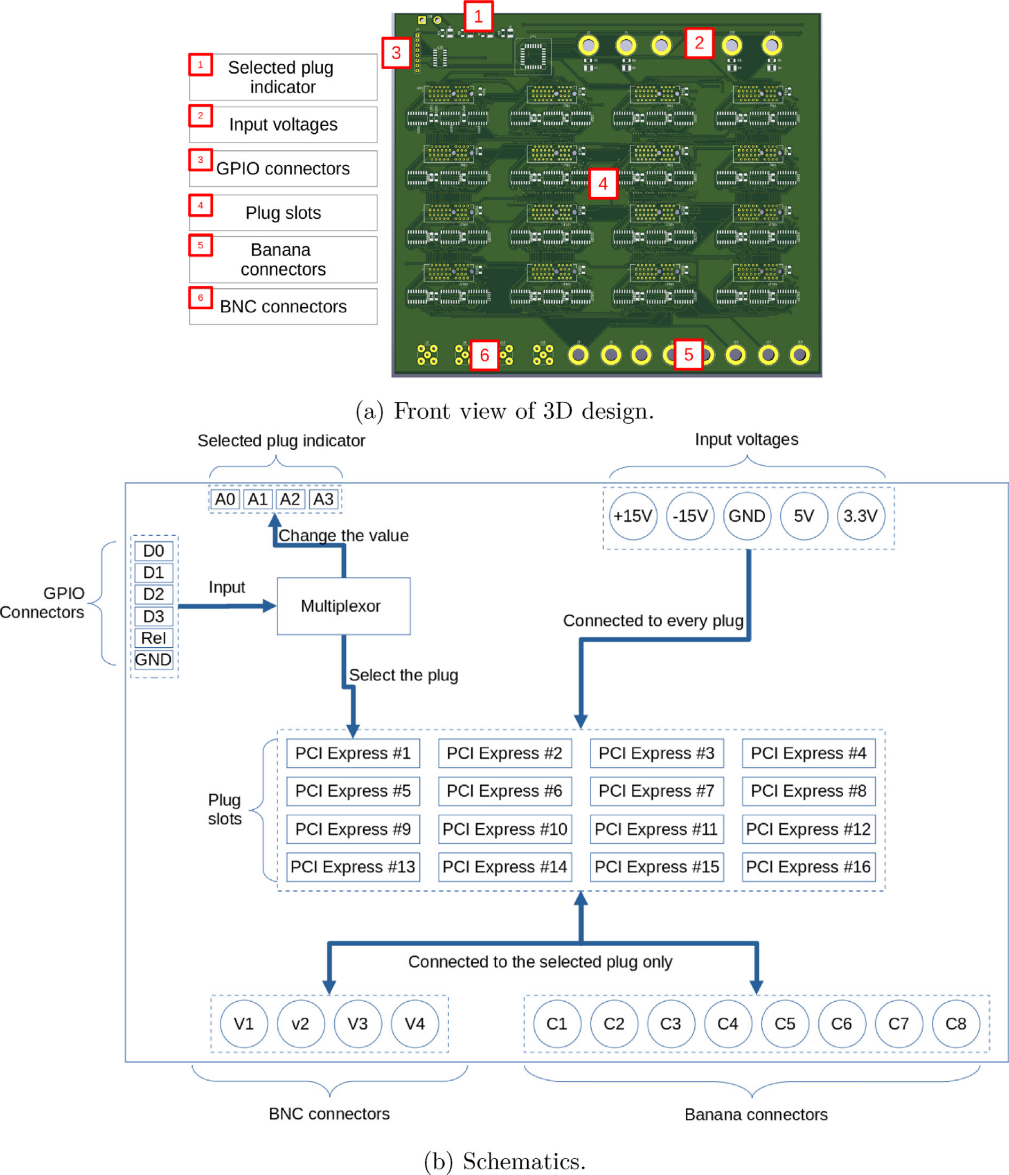
Motherboard.

A PCB called motherboard (see Fig. 5) is designed in order to:

• connect all the electronic elements of Laborem,
• provide power supplies (see Section 2.5) and and all useful signal (typically inputs/outputs) on each plug,
• design a robust and easy system to quickly change plugs (PCI Express slot).

The motherboard has 4 general-purpose input/output (GPIO) connectors which enables the selection of a specific plug to be studied among the 16 connected plugs. The plug, connected to a PCI Express slot (from 1 to 16), is selected via a multiplexer controlled by 4 bits from Raspberry Pi GPIO’s. 12 inputs/outputs (I/O) (4 BNC $V_{1}$ to $V_{4}$, and 8 banana connectors $C_{1}$ to $C_{8}$) are also available to easily connect other signals from peripheral instruments such as oscilloscope, multimeter, function generator, robotic arm, or relays (see Fig. 10, Fig. 17 for I/O connections). The signal voltages are limited between −15 V and +15 V with a maximum intensity of 1A.

### 2.4 Plugs

A plug is an electronic card intended to be connected to the motherboard. The currently available plugs enable the study of 4 passive filters (low-pass, high-pass, Wien band-pass, and band-rejection filter) and 4 active filters (low-pass, high-pass and band-pass of Sallen-Key type, plus another second order band-pass) with or without robot. All schematics are available in Section 3.

**Fig. 6:**
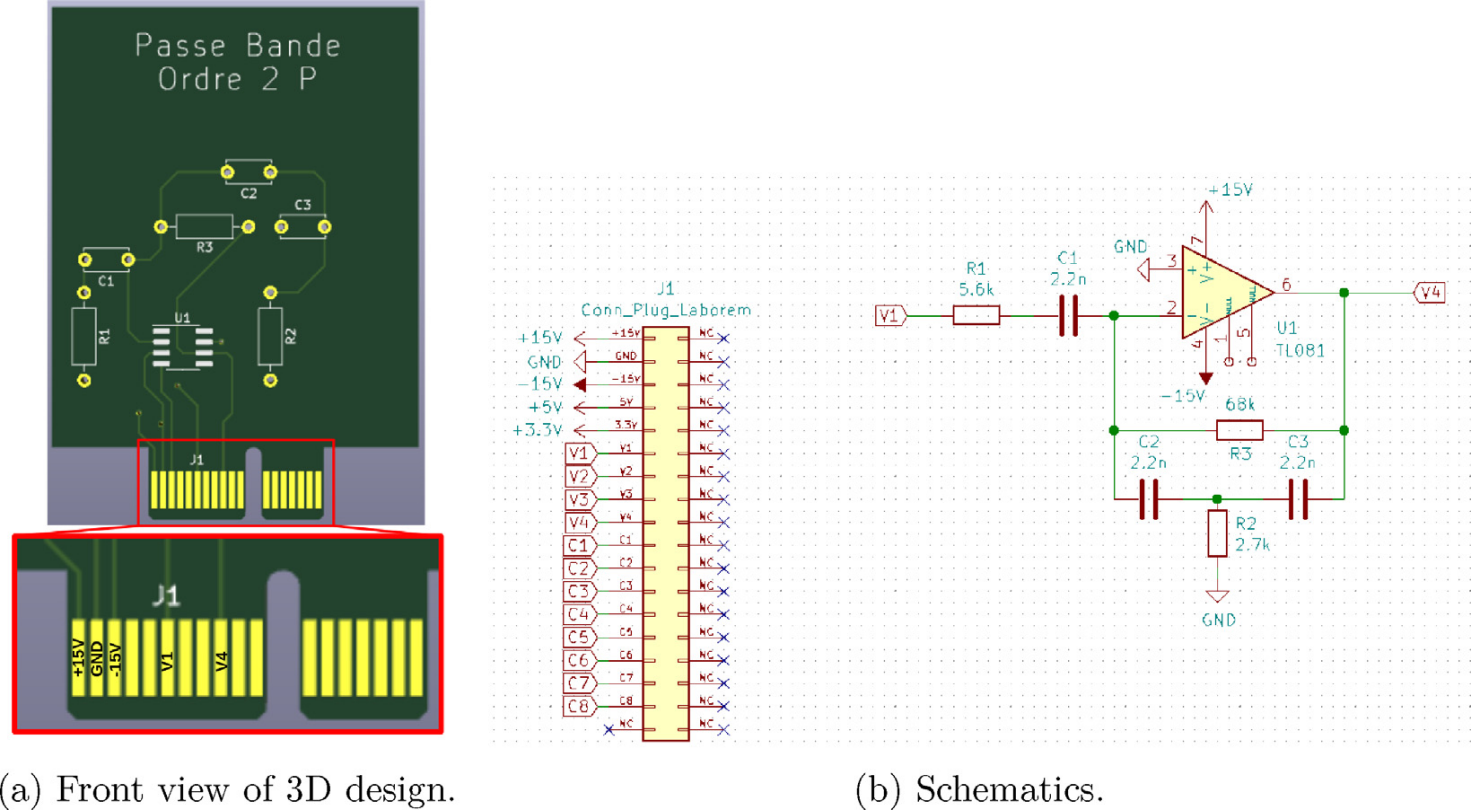
Pre-wired band-pass filter of order 2.

An example of a pre-wired band-pass second order active filter is given in Fig. 6. Components of the schematics shown in Fig. 6(b) are:

• 1 operational amplifier: TL081
• 3 resistors: 68 kΩ, 2.7 kΩ and 5.6 kΩ
• 3 capacitors: 2.2 nF
• the PCI Express connector shown in Fig. 6a

The input is connected to $V_{1}$ and the output is connected to $V_{4}$.

**Fig. 7:**
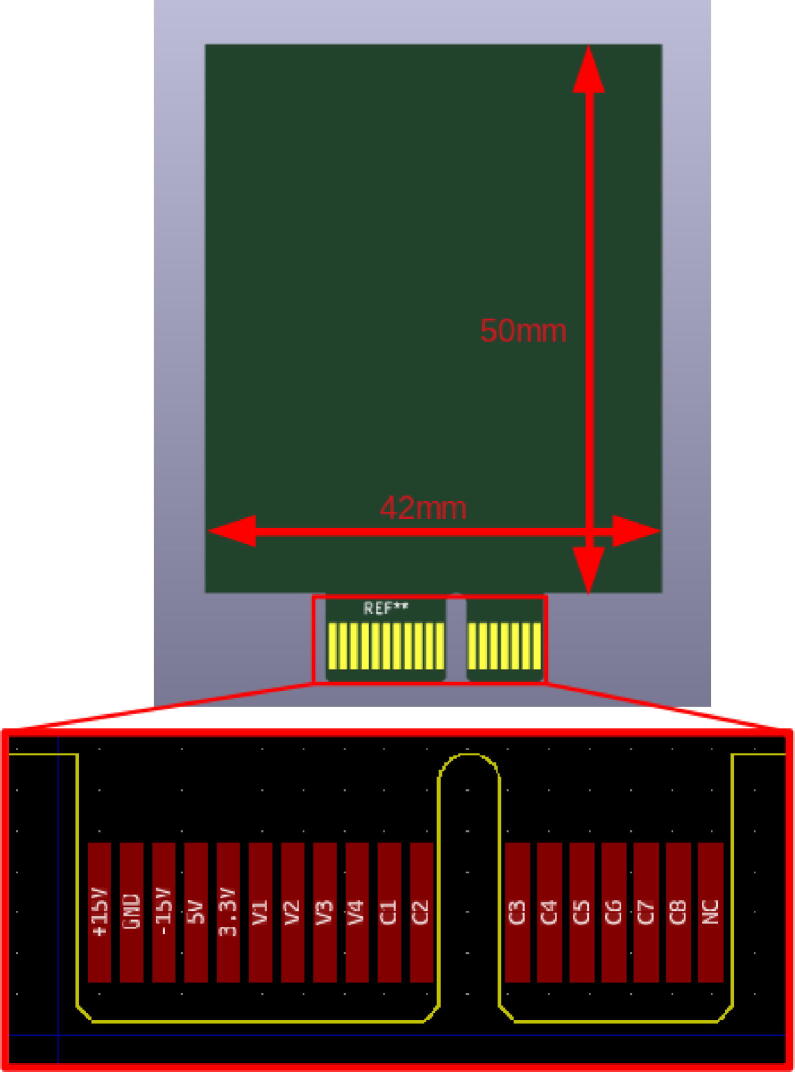
Detail of the PCI Express port connections on a plug.

All supply voltages and ground (see Section 2.5), as well as the 12 I/O connectors, are routed on the motherboard to each plug (see Fig. 7). The I/O connectors are relayed and only connected to the selected plug by the multiplexer of the motherboard.

In order to design a plug, the available space on the board is the limiting factor:

• 42 mm wide.
• No height limit, theoretically. Plugs up to 50 mm high are currently used.

Two types of plugs have been designed:

• pre-wired: all components of the circuit under study are present
• robot-wired: two components of the original circuit are missing and the student must use a robot to choose and place these two components on a board connected to the plug via I/O ports (typically $C_{1}$-$C_{2}$ for the first component and $C_{3}$-$C_{4}$ for the second one) on the motherboard

### 2.5 Power supply card

**Fig. 8:**
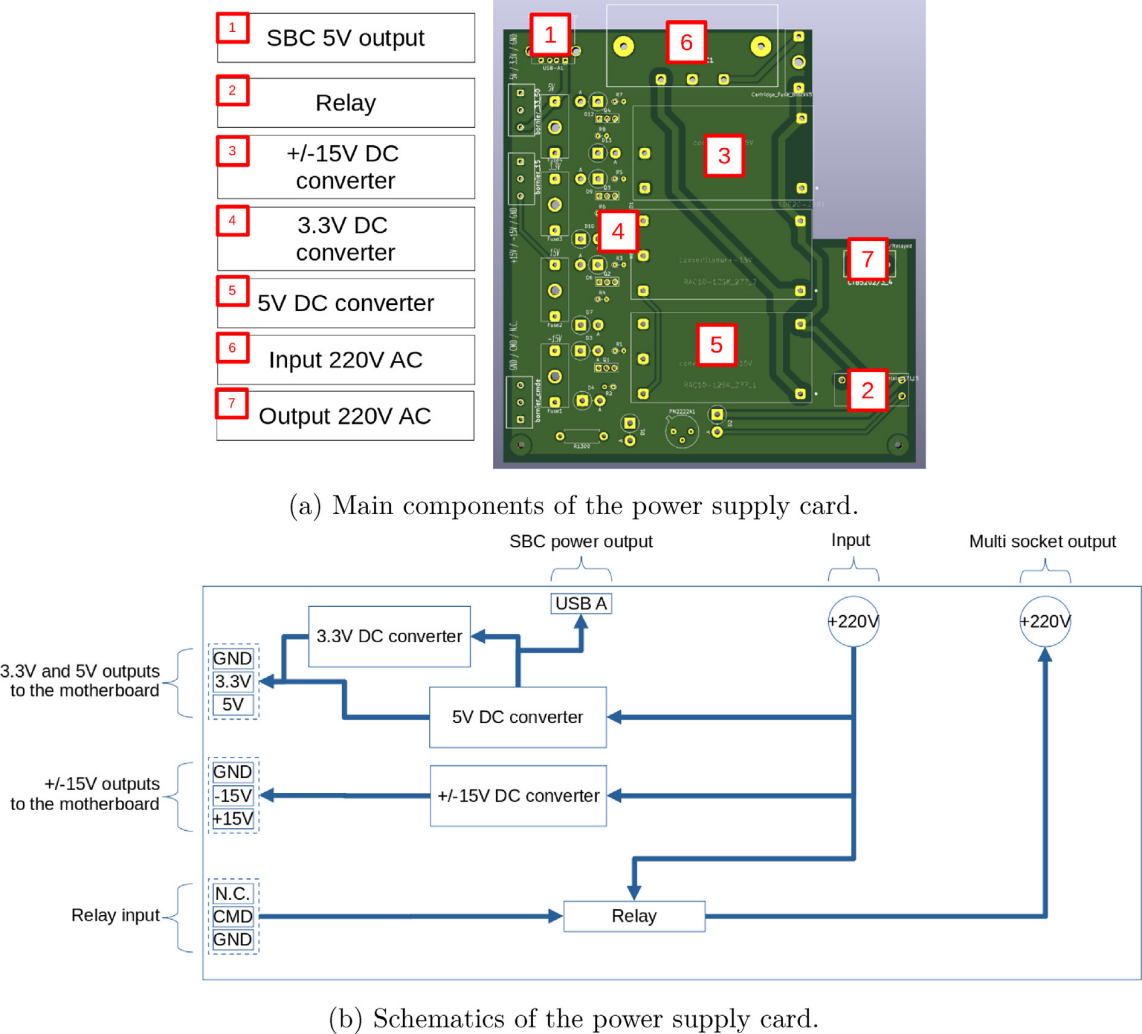
The power supply card.

The power supply card (see Fig. 8) that is powered by a main socket (220 V AC) provides DC voltage to the Raspberry Pi (5 V, 3A) and DC voltages (+/-15 V, 5 V, 3.3 V) to the motherboard and the connected plugs. Moreover, the power supply board is equipped with a relay that enables to switch off the power supply of a multi-socket where the external instruments are connected (oscilloscope, multimeter, function generator, robot) in order to save energy when the platform is not used by a student. All output voltages are protected by fuses.

## 3 Design files summary

Table 2 shows all the files available in our Zenodo repository^5^ necessary to build the PCB and the 3D box.

• PowerSupplyCard.zip: It contains Kicad source files which enables card modifications as well as Gerber files which enables the manufacturing of the card.
• 3DBox.zip: It contains Solidworks files which enables the modification of the box as well as STL files which enables the manufacturing of the box.
• Motherboard.zip: It contains Kicad source files to modify the board and Gerber files to build the board.
• Plugs.zip: It contains Kicad source files to modify the board and Gerber files to build the board.

**Table 2:** Design files summary.

Design filename	File type	Open source license	Location of the file
PowerSupplyCard.zip	Kicad and Gerber files	GNU GPL v3	Zenodo repository^a^
3DBox.zip	Solidworks and STL files	GNU GPL v3	Zenodo repository^a^
Motherboard.zip	Kicad and Gerber files	GNU GPL v3	Zenodo repository^a^
Plugs.zip	Kicad and Gerber files	GNU GPL v3	Zenodo repository^a^

a doi.org/10.5281/zenodo.5564369

## 4 Bill of materials summary

Table 3 shows the categories in the Laborem Box bill of materials and the associated total cost. Necessary components for each category can be found inside bill of material.^6^

**Table 3:** Bill of materials summary.

Designator	Component	Number	Cost per unit - €	Total cost - €	Source of materials	Material type	
Motherboard	Sheet Motherboard in BOM.ods^a^	See in BOM.ods^a^	See in BOM.ods^a^	205.32	See in BOM.ods^a^	Non-specific	
Power supply card	Sheet PowerSupplyCard in BOM.ods^a^	See in BOM.ods^a^	See in BOM.ods^a^	92.84	See in BOM.ods^a^	Non-specific	
3D box	Sheet 3Dbox in BOM.ods^a^	See in BOM.ods^a^	See in BOM.ods^a^	101.13	See in BOM.ods^a^	Non-specific	
Plugs	Sheet Plugs in BOM.ods^a^	See in BOM.ods^a^	See in BOM.ods^a^	29.74	See in BOM.ods^a^	Non-specific	

a https://doi.org/10.5281/zenodo.5564369

## 5 Build instructions

The assembly of a Laborem Box is divided into 6 steps:

• soldering all of the components on the motherboard,
• soldering components on the plugs,
• soldering components of the power supply board,
• printing the 3D box,
• assembling the different components in the box,
• connecting the instruments,
• deploying the software.

### 5.1 Soldering

Solder all components of the motherboard, plugs, and power supply board. It is easier to start with the surface-mount components (SMC). There are no SMC components on the power supply card. The only SMC components on plugs are the operational amplifiers (TL081). Through-hole technology components on the motherboard are all of the I/O components (banana, BNC, PCI Express, terminal blocks and pin connectors).

### 5.2 Box printing

STL files, necessary for printing, were generated from the Solidworks software. Printing on our 3D printer takes about 20 h.

### 5.3 Assembling

**Fig. 9:**
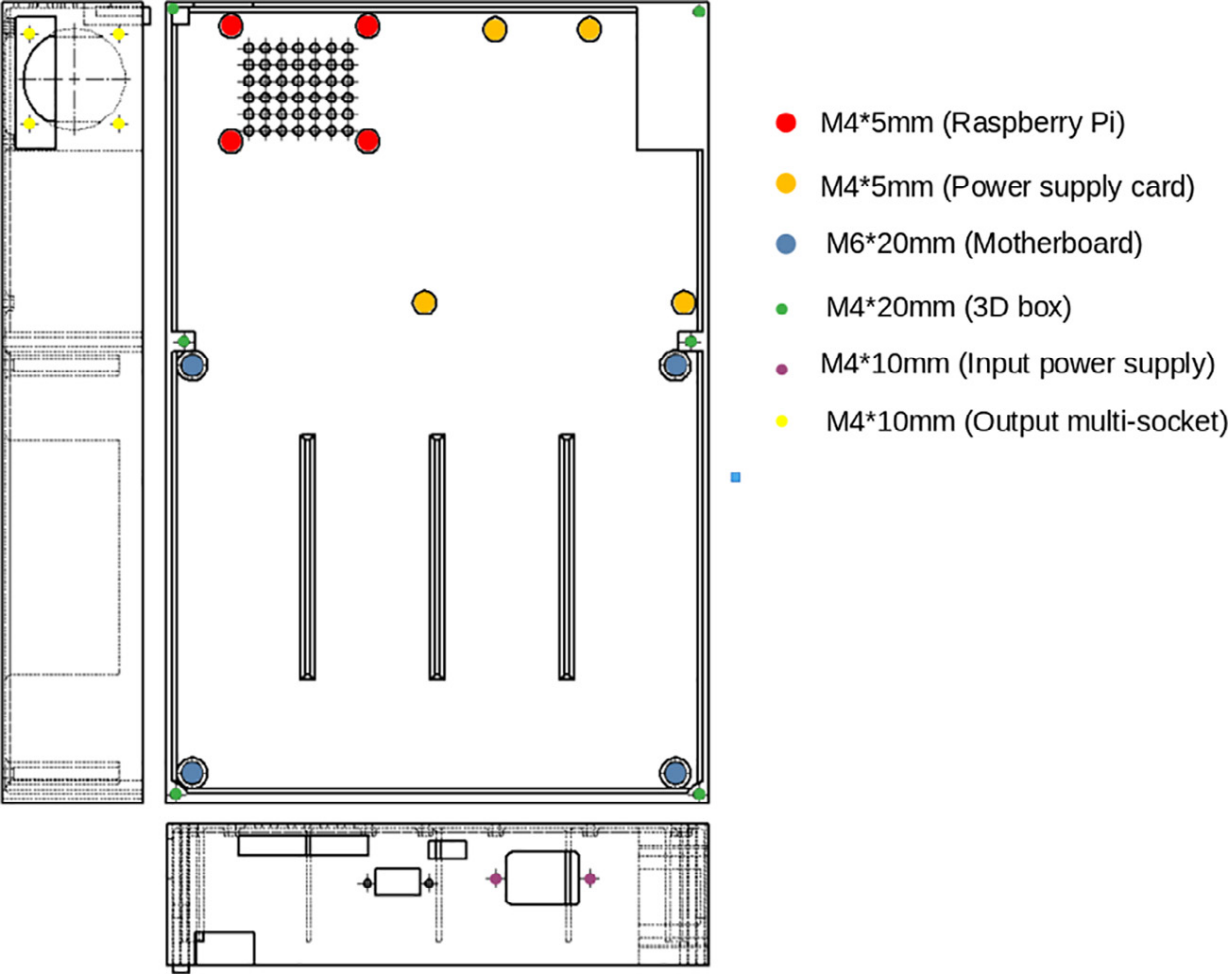
Locations of screws used in assembly.

Refer to Fig. 9 to use the correct screws to assemble the motherboard, power supply board, and Raspberry Pi in the Laborem Box.

**Fig. 10:**
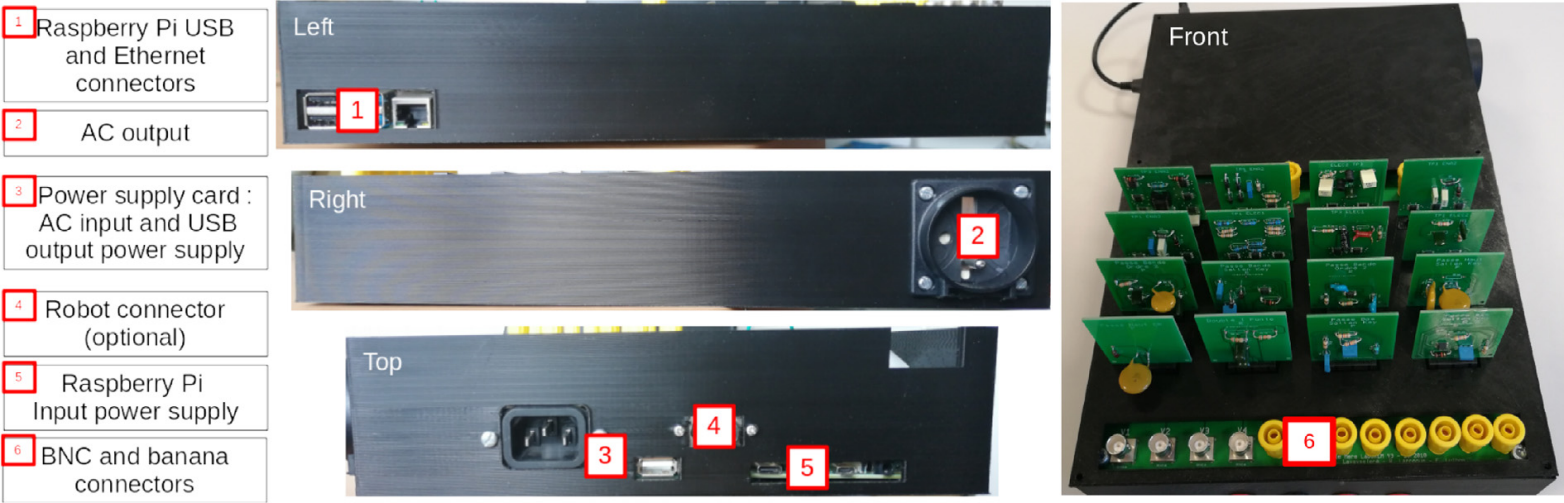
Laborem Box I/O.

I/O communication ports of the Raspberry Pi (USB, Ethernet, HDMI, power) must be accessible from the outside (see Fig. 10). The Ethernet connector should be located on the left side when the Raspbery Pi is on the top left of the Laborem Box.

For the power supply card, the input and the USB socket should be located on the top where it is easily accessible. The power supply input must be correctly fixed on the side of the box (see Fig. 10).

The output socket for the power strip must be connected to the cables before it is attached to the box (see Section 5.4 and Fig. 10).

### 5.4 Connecting

**Fig. 11:**
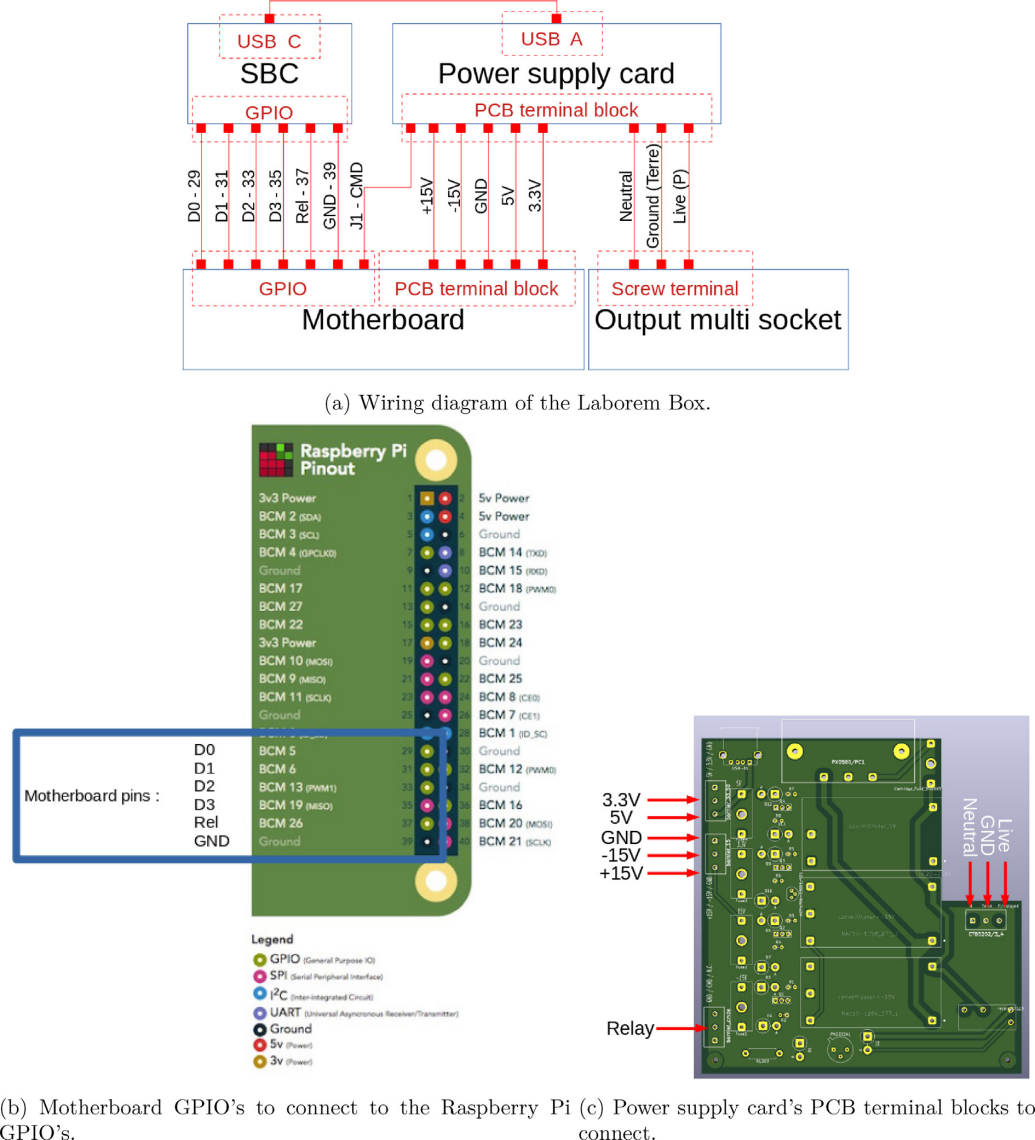
Wiring instructions.

Wiring diagram of the Laborem Box is shown in Fig. 11a.

In order to select a plug via the nano-computer, simply connect terminals D0-D1-D2-D3-Rel-GND of the motherboard to terminals 29–31-33–35-37–39 of the Raspberry Pi (see Fig. 11b).

For the power supply card (see Fig. 11c):

• Connect output for power supply strip to the PCB terminal block (bornier_3_points with N for neutral, Terre for ground, P for live) of the power supply board.
• Connect power supplies from motherboard to power supply board via the riser clamps (bornier_15 with  + 15 V, −15 V and GND, bornier_33_50 with 5 V and 3.3 V).
• Connect the relay control from motherboard (J1, left) to PCB terminal block of the power supply board (bornier_cmde with CMD)

Power the Raspberry Pi connecting USB A power supply to the Raspberry Pi USB C power using an USB cable (see Fig. 10).

### 5.5 Deploying

A SBC such as a Raspberry Pi or any computer with a GPIO additional card can be used.

A software named PyScada, for Python and SCADA (Supervisory Control And Data Acquisition) is currently used. The purpose of this software is to receive data from different sensors or instruments and control them via a simple web page. PyScada does not require any plugin to be accessible from a modern browser. Alerts and scripts make the system interactive and smart. An administration panel enables the system to be configured in order to build the user panel for students by inserting text, values, buttons, forms, graphics, images, and live videos.

The software runs under Linux distribution Debian and its derivatives (to install Raspi OS on a Raspberry Pi see [22]). Currently, PyScada has not been tested on Mac OS nor Windows. However, as it is written in Python, HTML, Javascript and CSS, it is easily portable to other operating systems.

To install PyScada and Laborem, one can use an automatic installation script. To do this, the protocol given below has to be followed:

• Connect to the Raspberry Pi with ssh^7^.
• Download the installation script:
  wgetht tps : / / raw . g i thubus e r c ont ent . com/ clavay /PyScada?Laborem/master / e x t r a s /i n s t a l l . sh−O i n s t a l l . sh
  sudo chmod a+x i n s t a l l . sh
  sudo . / i n s t a l l . sh
• During the installation script:
  - Specify whether the Raspberry Pi uses a proxy to access the Internet.
  - Choose to install PyScada from the clavay fork
  - Choose to install PyScada-Laborem, PyScada-GPIO, and PyScada-Scripting
  - You will need to enter the root password of the Raspberry Pi (’raspberry’ by default)
  - You will create the first PyScada user (’pyscada’ by default)
• Install the database:
  - Download laboremDB.json from Zenodo repository 8.
  - sudo -u pyscada python3/var/www/pyscada/PyScadaServer/manage.py loaddata laboremDB.json

## 6 Operation instructions

### 6.1 Risks

The Laborem Box should NEVER be opened while anything is physically connected to the box.

To avoid corrupting the Raspberry Pi’s database, it is best to turn it off before disconnecting the box.

### 6.2 Connect to the interface

To use the hardware, the easiest solution is to use the software we developed. You need a web access to the Raspberry Pi (through an ethernet or wifi network, for example see [23]).

• Connect to http://raspberryPi_IP_address/
• Use the credentials you enter during the installation of PyScada.
• Add your user in the teacher group:
  - click on your username (see Fig. 15)
  - click on “Admin”
  - click on “User” and find your username
  - in the group list add “teacher”
  - save changes.

### 6.3 Test the motherboard’s connections

**Fig. 12:**
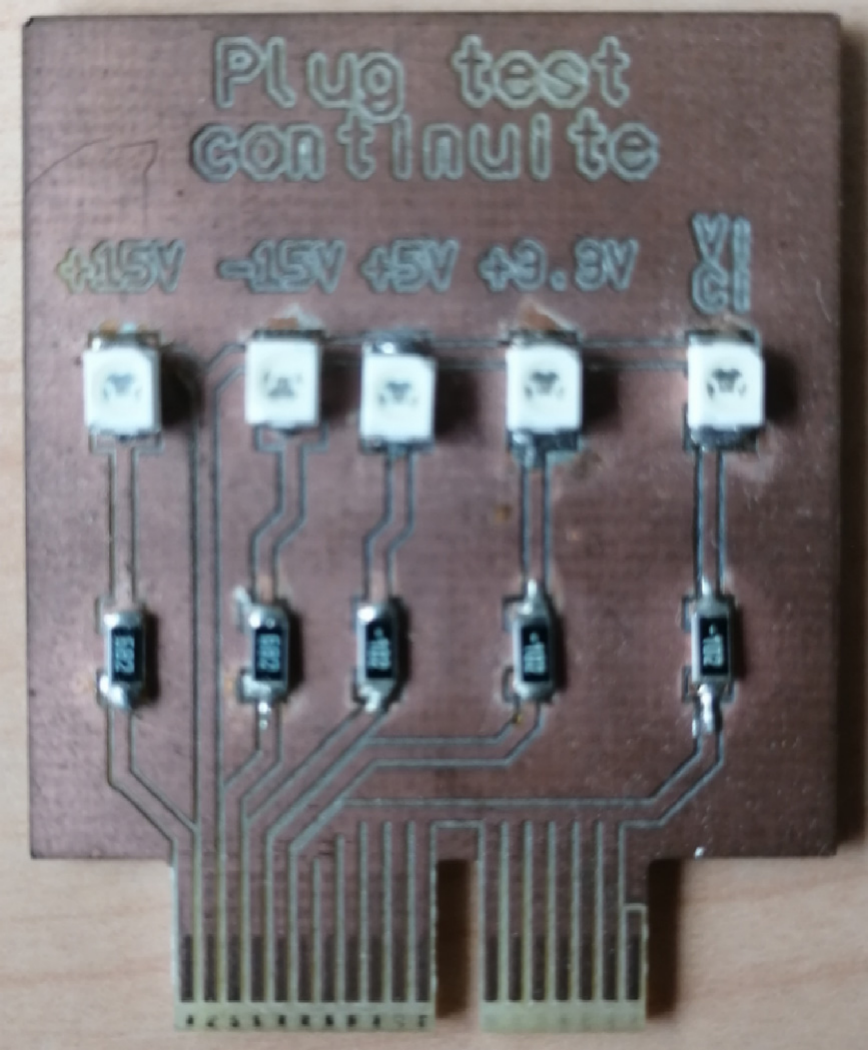
Test plug used to check the motherboard connections.

A test plug (called “plug continuite” in Plugs.zip from Section 3) which enables to check a part of the soldering of the motherboard has been developed (see Fig. 12). The test plug has 5 LEDs, one for each supply voltage (see Section 2.5) and and one for all the *V* and *C* I/O (see Section 2.3).

It is therefore possible to check if power supplies from the motherboard reach each PCI Express terminal block by plugging in the test plug and confirming that each 4 left-hand LEDs are illuminated.

Since the *V* and *C* I/O connections are only connected to the plug selected by the motherboard, it is necessary to:

• select the plug slot where the test plug in the PyScada interface is connected,
• connect the 5 V to each *C* and *V* I/O using a banana cable,
• see the last LED light up for each input connected to the 5 V.

**Fig. 13:**
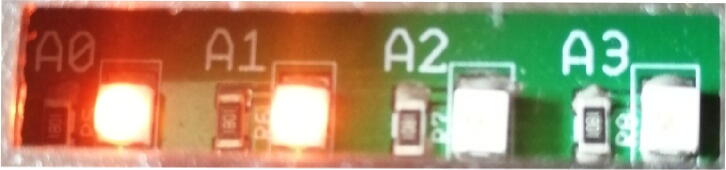
4 LEDs on the motherboard indicate the selected plug.

LEDs located on the motherboard next to the GPIO connectors enable the user to check the selected plug by transforming the value (on  = 1, off  = 0) of the binary word (A3 A2 A1 A0) into decimal. Example: in the Fig. 13, the motherboard indicates decimal value of 3 that correspond to the plug 4 being selected (decimal values from 0 to 15 corresponds to the plugs from 1 to 16). Any teacher can manually change the (A3 A2 A1 A0) value (see Section 6.5).

### 6.4 Student interface

This interface is only available in French for the moment. Laborem does not implement a reservation system. As Laborem access is available via Moodle Learning Management System (LMS) (see Section 7.1), it is possible to schedule the access to Laborem for each student. However, a direct access can be done by typing the local URL in a web browser. In order to manage several students connected at the same time on the platform, a queue management has been computed. There are three status for the student in the queue:

• Worker: the first student in the queue and the only one who can access the instruments. As soon as another student appears in the queue, the worker is given a limited amount of time before handing over to the next student in the queue.
• Viewer: this status only enables to see what the worker is doing (for example: instruments configuration, curves visualization).
• Waiter: the student can only knows his rank in the queue and the time needed to wait before accessing the devices.

**Fig. 14:**
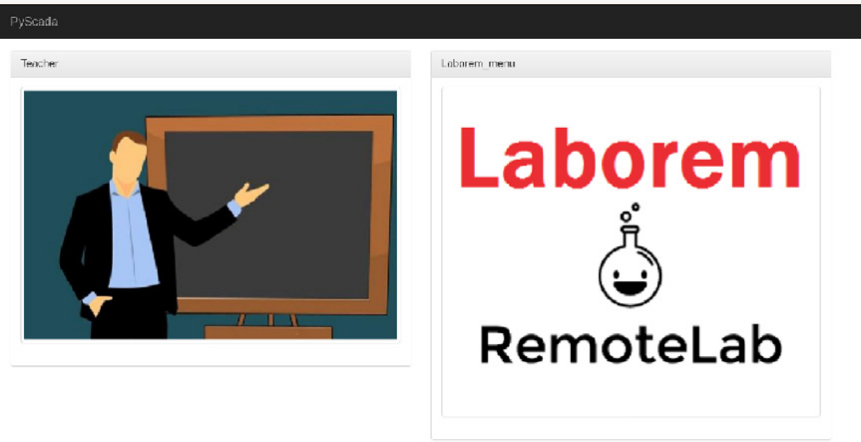
Access to the teacher and student interfaces.

In order to enable collaborative work, the teacher can define a limited number of viewers. To enter the student interface, simply click on the image “Laborem RemoteLab” (Fig. 14).

**Fig. 15:**
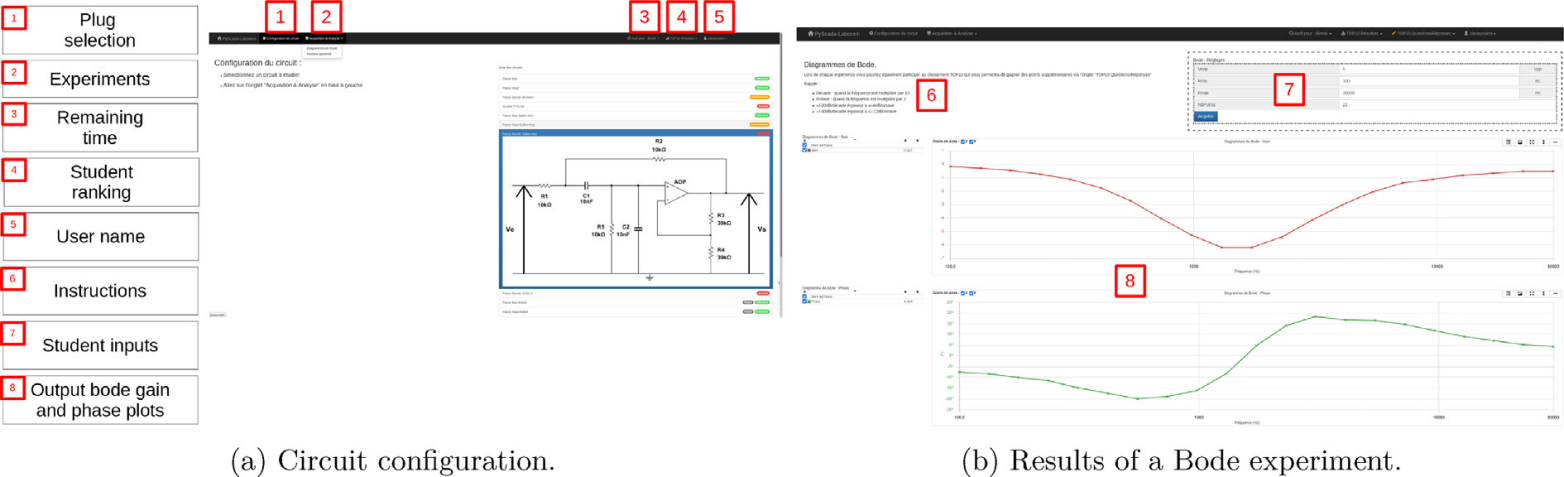
The easy-to-use student interface.

Student interface is composed of 3 parts, accessible via the navigation bar (see Fig. 15):

• Home: explain the aim of the remote practical work, as well as the waiting queue in case other students are trying to access to the platform simultaneously.
• Plug selection: choose the plug to be studied.
• Experiments: provide the list of experiments available, such as studying Bode diagrams or analyzing the response signal of a filter.

Students are invited to answer questions when they perform an experiment, and they can also compare themselves to others via a ranking (which as been designed to look like a “hall of fame” part of a game [20]). These menus are available via the navigation bar.

### 6.5 Teacher interface

The teacher’s interface is only visible and accessible to users who are members of the teacher’s group. The list of users in the group can be edited in the administration interface (see Section 6.2). It is accessible by clicking on the teacher’s image (see Fig. 14). Teachers can use this interface to change the duration of the access for a student to the platform, to restart instruments connected to the power strip, and to change the selected plug with (A3 A2 A1 A0) (see Section 6.3).

### 6.6 Administration interface

The administration interface is accessible to users defined as “staff”. By default, the user “pyscada” is staff and can add other members. The user password of this user is defined during the installation (see Section 5.5).

The administrator interface enables access to the whole configuration of PyScada:

• edit student and teacher HMI (PyScada HMI section)
• edit scripts handling the experiments (PyScada Scripting section)
• modify instruments connected to the motherboard (PyScada Core section)
• modify the courses studied and the subsequent list of plugs that appears on the student interface (PyScada Laborem section)
• edit set of questions asked and the student’s answers (PyScada Laborem section).

To connect to the administration interface with an account that has access rights, click on your username at the top right of the navigation bar (Fig. 15) and and then on “Admin”.

## 7 Validation and characterization

### 7.1 Add your own electrical circuits for your students

**Fig. 16:**
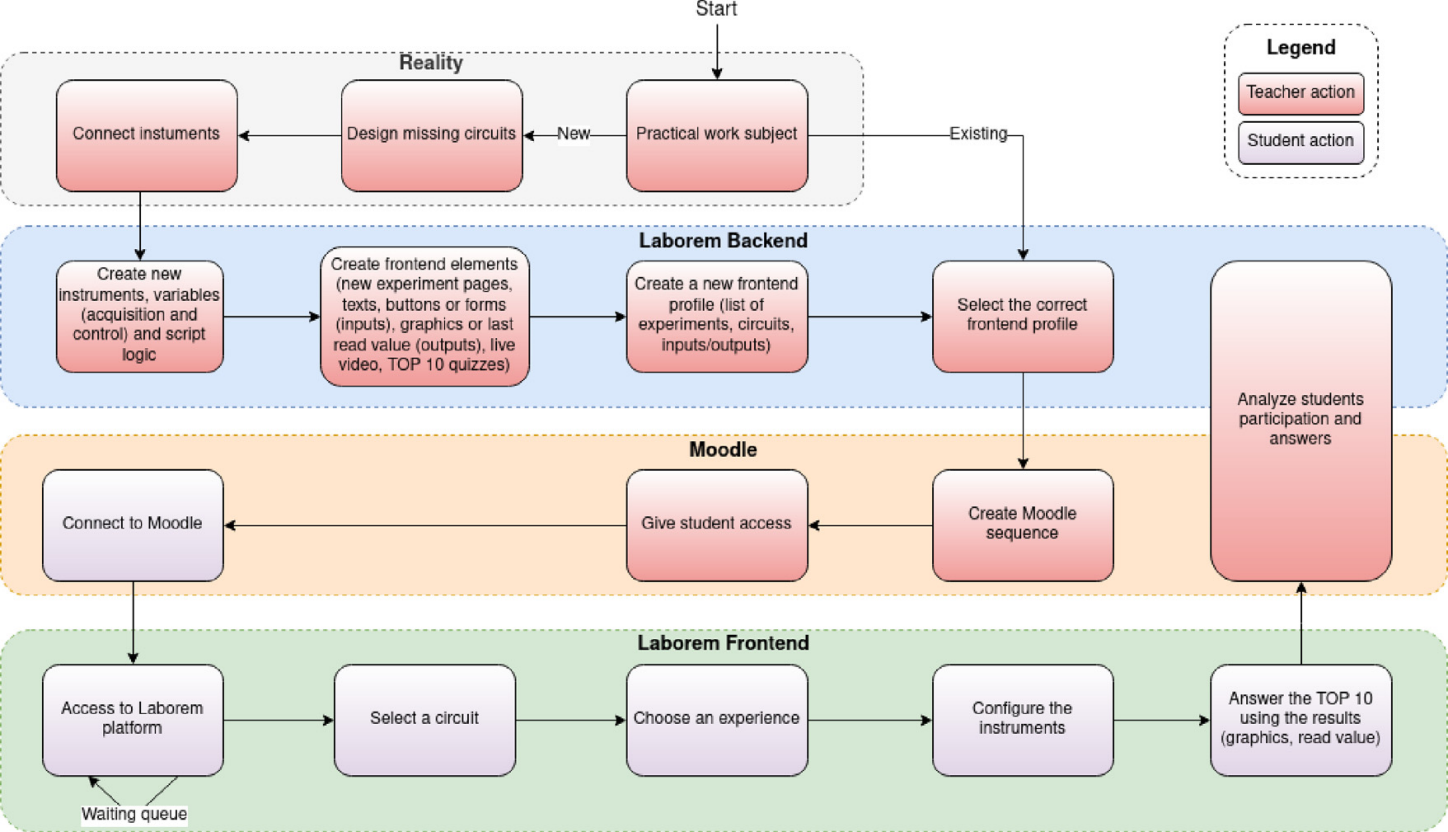
Laborem process including Moodle integration.

The Laborem Box was designed to enable a teacher to create and use their own electrical circuits. The Fig. 16 details the process of Laborem integrated into Moodle^9^. The teacher’s part consists of the following steps:

• create the material
• connect the instruments to the box
• configure the software:
  - create the instruments, variables, and logic in scripts
  - create the front-end elements
  - create or select the course profile.

#### 7.1.1 Design the circuit

First, draw your circuit from the blank plug available in the design files (Plug-template in Plugs.zip from Section 3) using the open source software Kicad^10^. You must respect the PCI Express terminal block presented in Fig. 7. For example, if you decide to connect channel 1 of your oscilloscope to $V_{4}$ and the output of your function generator to $V_{1}$, you will have to route the $V_{1}$ and $V_{4}$ terminals of the PCI Express connector to the corresponding place in your electrical circuit (as done in Fig. 6).

Then you have to order the PCB (such as from JLCPCB^11^) and the components (such as from Mouser^12^) and solder them.

#### 7.1.2 Connect the instruments

**Fig. 17:**
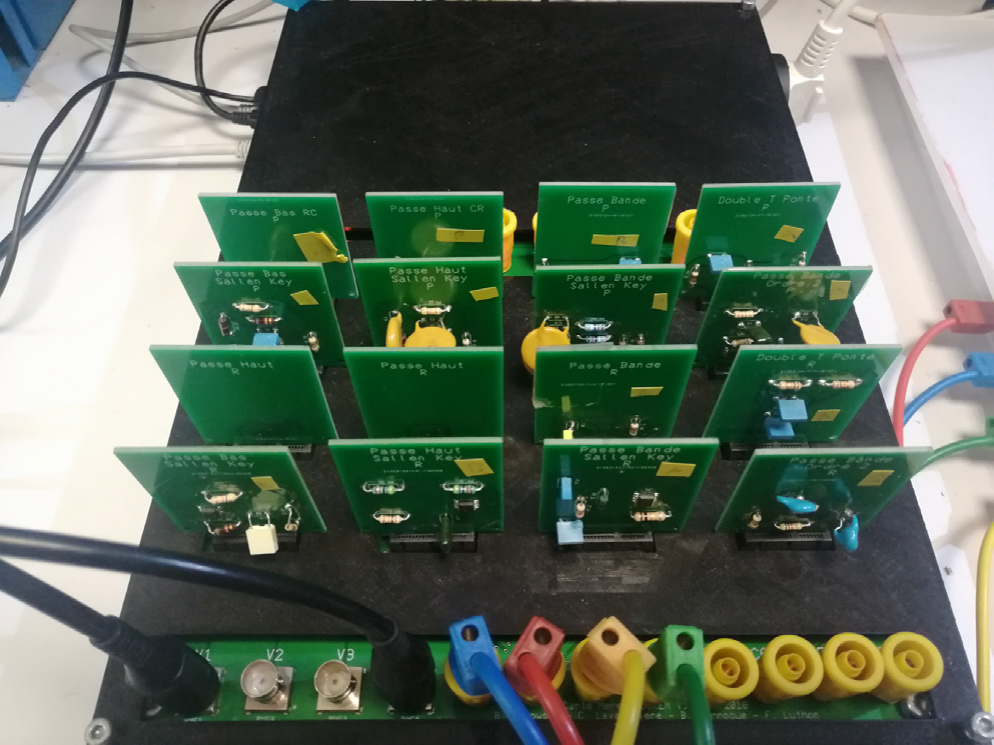
BNC, bananas and plugs connected to the Laborem Box.

Connect the instruments to the Laborem Box using BNC and banana connectors (Fig. 17) as defined during the circuit’s design. These signals are available on each plug when selected by the motherboard.

#### 7.1.3 Create instruments, variables, and script for the experiment

**Fig. 18:**
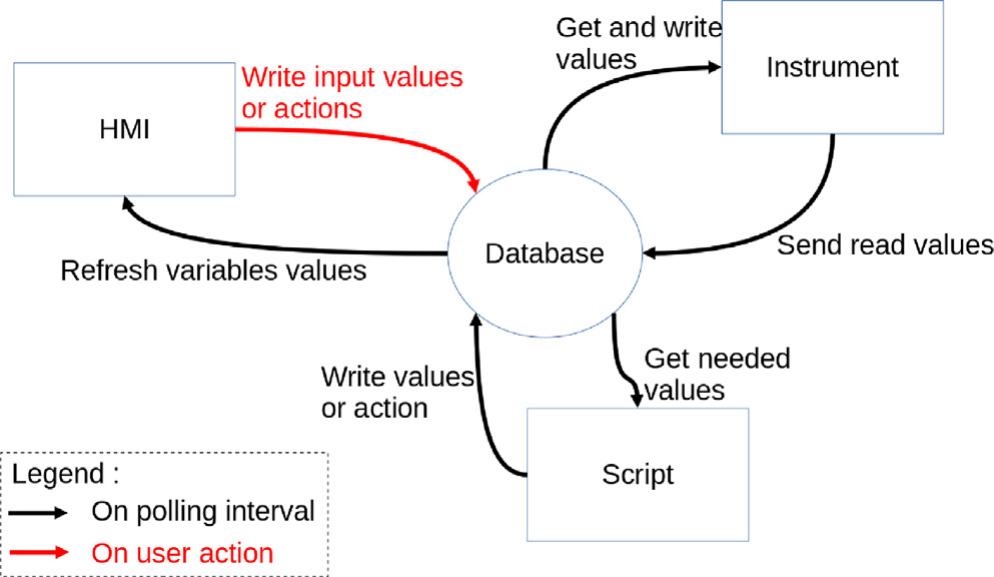
PyScada asynchronous logic centered on the database.

The Fig. 18 details the asynchronous execution of PyScada elements communicating via the database:

• each instrument has a polling interval defined to do an action:
  - get values (example: read a voltage from an oscilloscope)
  - write values (example: set the frequency of a generator)
• each script has a polling interval defined to do an action:
  - get values from the database (example: a student wants to start an experiment)
  - write values to the database (example: Bode plots)
• HMI:
  - refresh all values each 2.5 s
  - send user inputs (example: voltage, frequencies and number of points of a Bode plots)
  - force instruments to read values or scripts to run.

If the instruments have not been predefined, you must create them in the PyScada Core/Devices section. You must also create all of the variables (PyScada Core/Variables section) to be read or written on the instrument (for example a voltage, a current, a frequency).

The example of the script can be found in [24] which enables the visualization of Bode diagrams^13^. The script can be added to the PyScada Scripting/Script section.

**Fig. 19:**
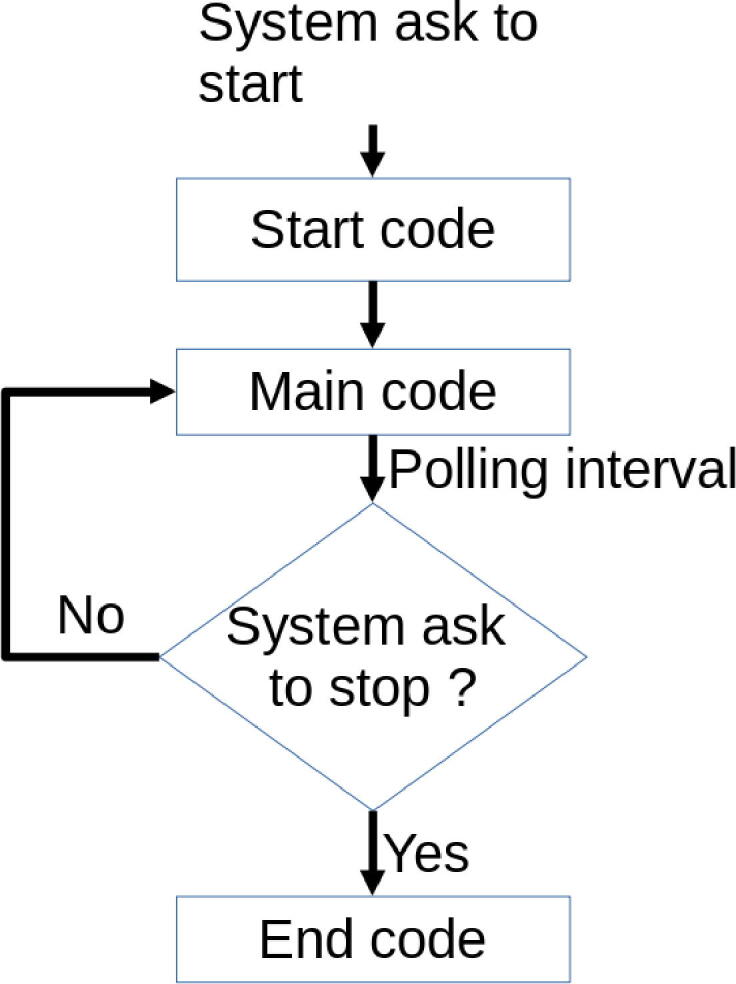
Script flow.

A script in PyScada is divided into 3 parts. One part is launched at startup, another at shutdown, and the main part at regular intervals as explained in Fig. 19.

At startup, the initialization of some variables for the student interface is needed: progress bar, stop button, reset of the displayed points, message indicating the system startup. Then the script checks that the motherboard has a selected I/O configuration.

When the script is stopped, it indicates to the student that Laborem has stopped and it doesn’t display any points anymore.

**Fig. 20:**
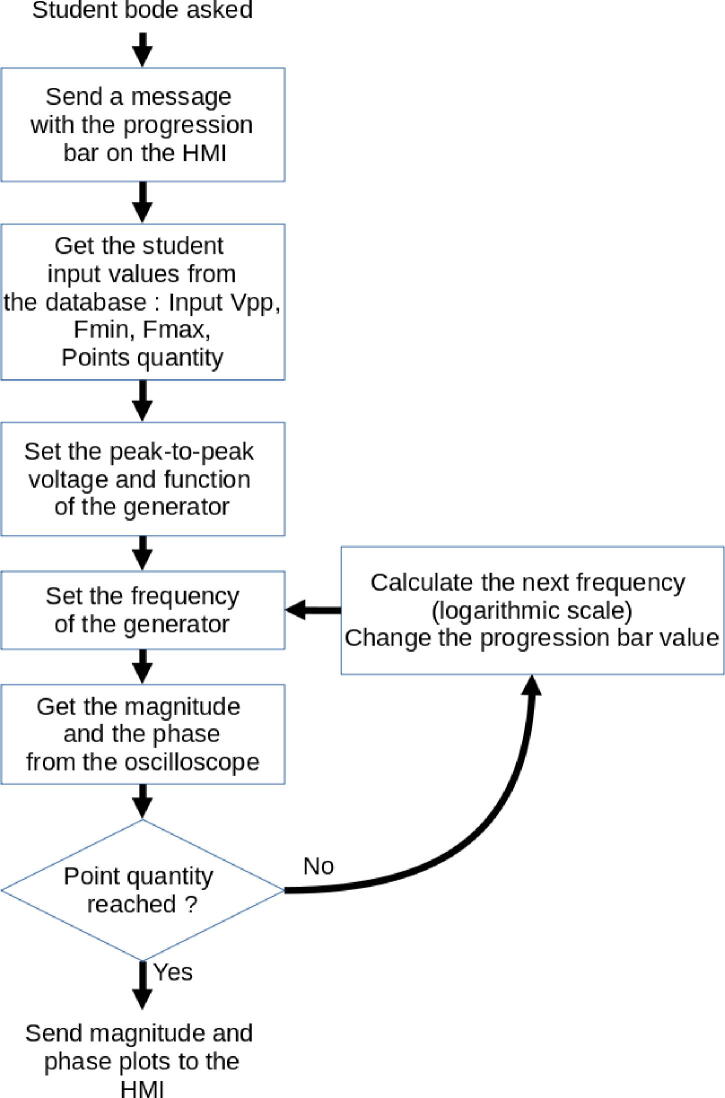
Bode script flow.

The main code scans every second if a Bode diagram has been requested by a student. The Fig. 20 details the different steps of the script enabling to make measurements and to display them to the student.

#### 7.1.4 Plug setup in PyScada

**Fig. 21:**
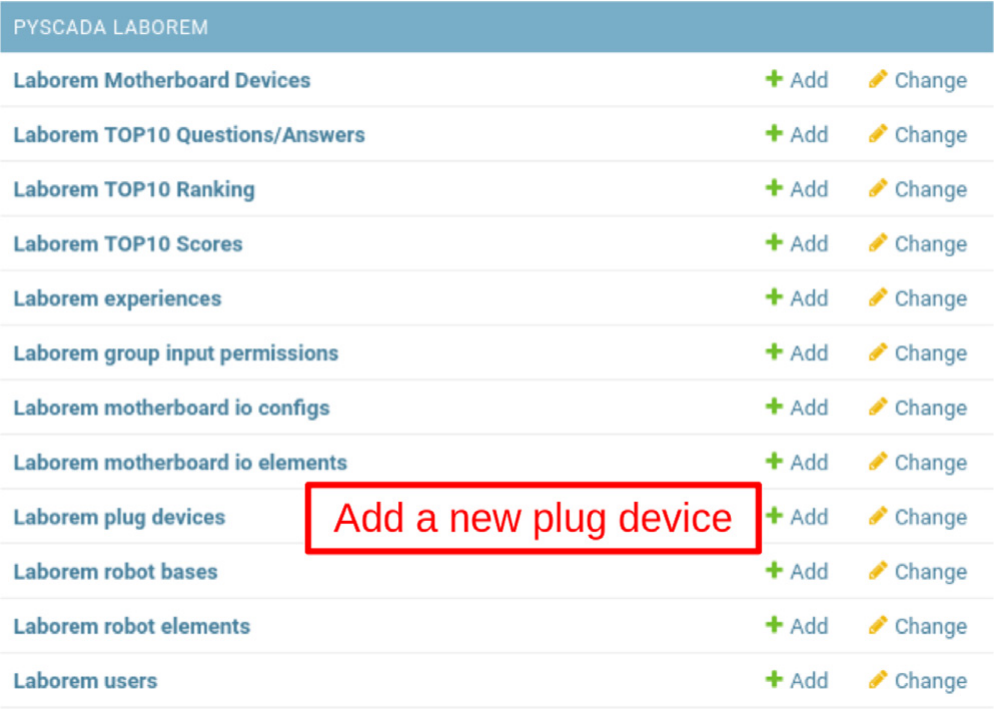
Add a new plug device in the administration interface.

In order to add the created plug to the list of available plugs, you just have to connect to the PyScada interface and go to the administration interface (see Section 6.6). In the “PyScada Laborem/Laborem plug devices” section, click on “Add” (see Fig. 21).

Add a name and a description, select an image with the circuit diagram.

You must choose an existing motherboard I/O configuration or create a new one (in our example: $V_{4}$ for signal output to be displayed on channel 1 of the oscilloscope and $V_{1}$ for signal input provided by the function generator). In the I/O configuration of the motherboard, you must indicate in which position the plug is located (from 1 to 16, see Fig. 5b).

**Fig. 22:**
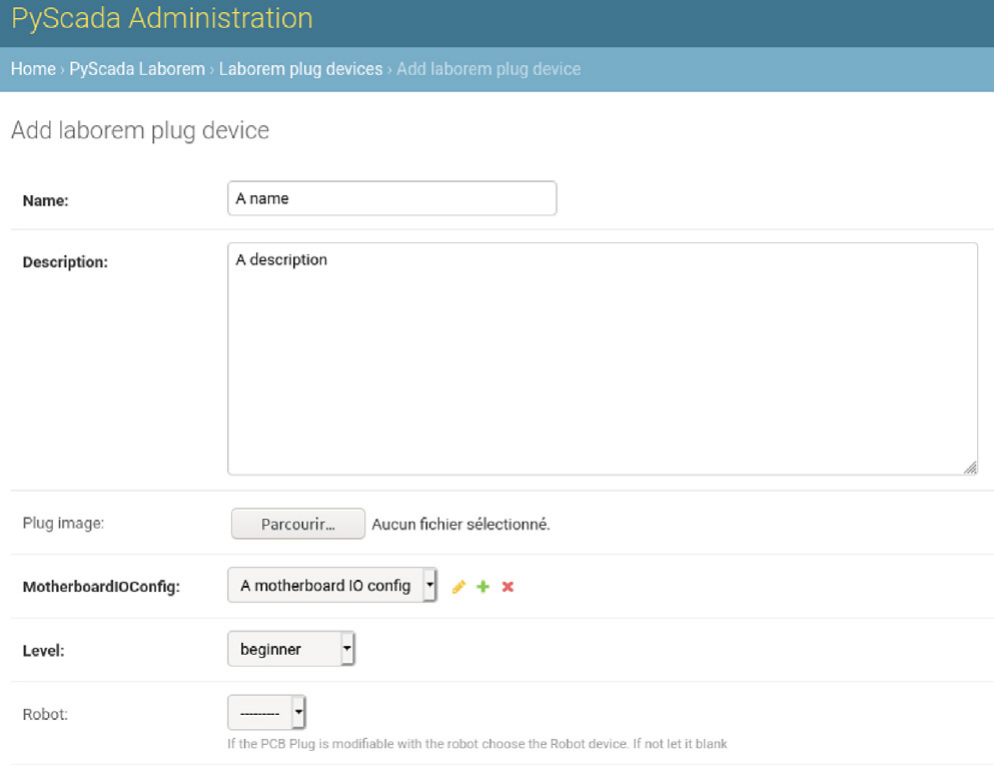
Fill in the new plug information.

Choose a difficulty level of the circuit (between beginner, intermediate, or advanced). If you use a robot, you must choose it in the “Robot” option (see Fig. 22).

**Fig. 23:**
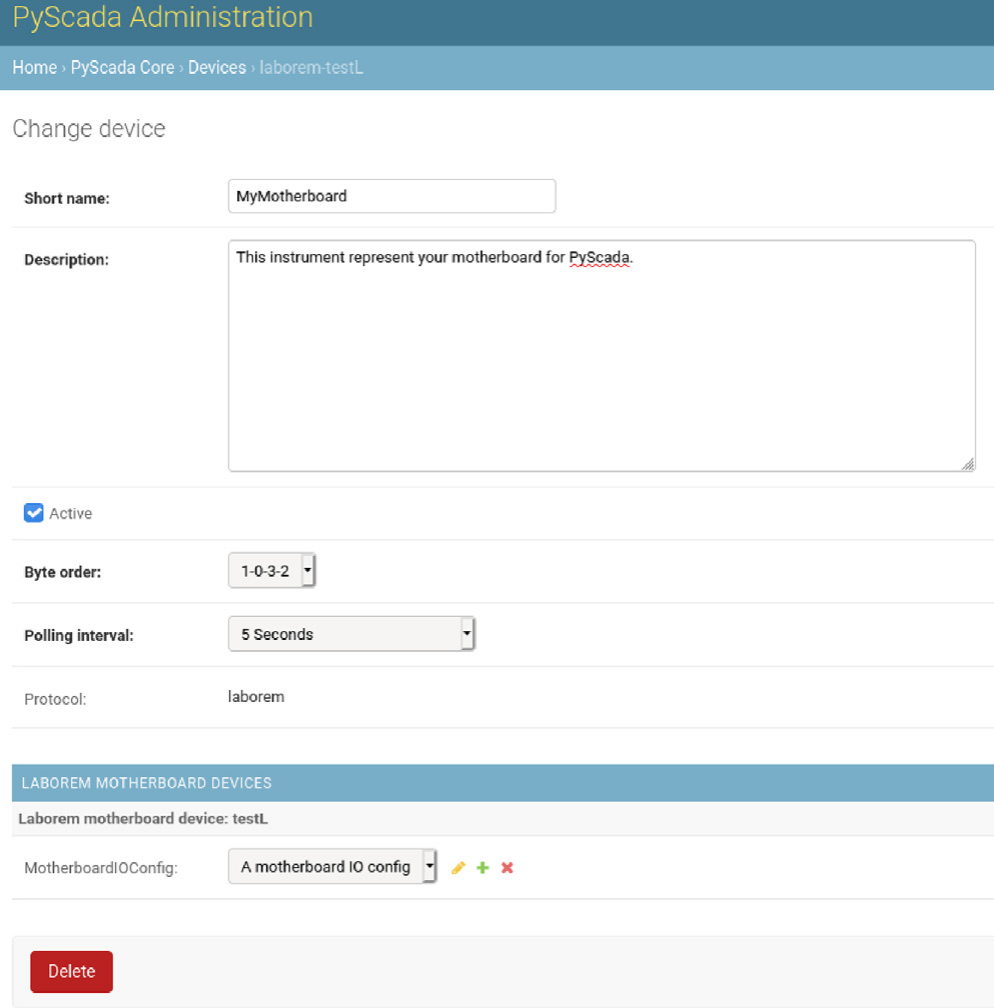
Select the right Motherboard I/O Config.

You must indicate in your motherboard configuration which I/O configuration you have connected (plugs with the same I/O configuration will be displayed in the student interface): in the administration interface, section “PyScada Core/Devices”, edit your motherboard, and select the correct I/O configuration as in the Fig. 23.

Go to the student interface in order to see the plugs defined in the selected motherboard I/O configuration displayed.

### 7.2 Analysis of students’ behavior regarding the use of open source remote laboratories

During the year of 2021, a class composed of 42 first-year undergraduate students from the IUT of Bayonne used the Laborem platform.

Students had to realize a practical work on active and passive filters. They had to study the frequency response of filters using Bode diagrams (gain and phase) as well as characterize the temporal response and the spectrum of signals.

In order to determine the effectiveness and satisfaction of Laborem, the class was divided into three groups to compare different use case scenarios (see [25] for details).

The influence of time management and pair learning was studied. By modifying two criteria, i.e., individual or paired passage and free or restricted time access, a comparison for each group was made:

• usage: frequency, duration and regularity,
• results: completion, grades, and pedagogical impact,
• satisfaction: motivation, feelings, expectations, and evaluation of the platform.

The first group had 14 days of free access to complete the practical work using Laborem. They had access to the platform at any time. The second group had one day reserved for each student, plus 7 days of free access. The last group was formed by pairs who had one day reserved for each pair, plus 6 days of free access.

Whether it is from the student’s point of view (motivation, feeling), the use (regularity, frequency, duration), or from the pedagogical results (score, completion), our study demonstrated the significant advantage of pair work in the use of a remote laboratory. In comparison, autonomous work with semi-restricted access in relation to free access did not bring a significant amount of added value.

### 7.3 Capabilities and limitations

Capabilities of Laborem are listed below:

• Student:
  - Carry out practical electronics work remotely.
  - Compare results with other students.
• Teacher:
  - Create new circuits to study.
  - Easily change accessible circuits.
  - Modify student interface.
  - Provide questions during experiment and analyze results.
  - Duplicate the platform.
  - Use the platform during class to do practical demonstrations.

Limitations of Laborem are listed below:

• To work in other fields: in order to propose practical work in other topics, it requires the development of specific plugs.
• Students cannot work on their previous measurements. Access to a history must be implemented.
• “Worker” student monopolizes the platform even if he/she does not access the instruments (see Section 6.4). In the future, in order to let all students access the HMI, the queue could manage the control of equipment to perform measurements.
• To have more than one circuit per plug. It is necessary to route on the motherboard some GPIO ports of the Raspberry Pi to the plugs slots in order to activate switches. This type of plug has been tested with manual wiring. Currently 4 circuits are inserted per plug.

## CRediT authorship contribution statement

**Camille Lavayssière:** Methodology, Software, Validation, Data curation, Writing – original draft. **Benoît Larroque:** Methodology, Resources, Writing – review & editing. **Franck Luthon:** Conceptualization, Supervision, Writing – review & editing.

## Declaration of Competing Interest

The authors declare that they have no known competing financial interests or personal relationships that could have appeared to influence the work reported in this paper.

## Footnotes

1 https://labsland.com/en/labs/electronics-community

2 http://online-engineering.org/SIG_visir.php

3 https://github.com/clavay/PyScada-Laborem/raw/master/extras/Laborem_robot.mp4

4 https://www.ivifoundation.org

5 https://doi.org/10.5281/zenodo.5564369

6 https://doi.org/10.5281/zenodo.5564369

9 https://moodle.org/

10 https://www.kicad.org/

11 https://jlcpcb.com

12 https://mouser.com

13 https://en.wikipedia.org/wiki/Bode_plot

## Footnotes

7 https://www.raspberrypi.com/documentation/computers/remote-access.html

8 https://zenodo.org/record/5564369
